# Quality Improvement Initiatives in Renal Biopsy for Patient-Centered Communication by Shared Decision Making

**DOI:** 10.3390/diagnostics12051227

**Published:** 2022-05-13

**Authors:** Cheng-Hsu Chen, Chia-Tien Hsu, Ming-Ju Wu, Shang-Feng Tsai

**Affiliations:** 1 Department of Post-Baccalaureate Medicine, College of Medicine, National Chung Hsing University, Taichung City 40227, Taiwan; cschen920@vghtc.gov.tw (C.-H.C.); wmj530@vghtc.gov.tw (M.-J.W.); 2 Division of Nephrology, Department of Internal Medicine, Taichung Veterans General Hospital, Taichung City 40705, Taiwan; jatenhsu@gmail.com; 3 Department of Life Science, Tunghai University, Taichung City 40704, Taiwan; 4 School of Medicine, National Yang-Ming University, Taipei City 11265, Taiwan

**Keywords:** quality improvement (QI), renal biopsy, patient-centered communication, shared decision making (SDM), patient decision aid (PDA)

## Abstract

Background: Renal biopsy is the gold standard for diagnosing renal disease. The major concern is bleeding. Shared decision making (SDM) has been reported to improve communication but has never been used regarding renal biopsy. Methods: We launched a 5-year project on SDM for renal biopsy. We collected cases of renal biopsy and bleeding, and cases of SDM. The process of quality improvement (QI) for SDM is also shared. Results: Taichung Veterans General Hospital has the largest number of renal biopsy cases, and the lowest bleeding rate in Taiwan. We enlisted a core team for this QI project and conducted stakeholder mapping. In 2017, we conducted a small pilot study for SDM based on printed material as a decision aid. The satisfaction rate was 95.5%. From 2018 to 2019, we improved SDM patients’ decision aid from printed material to four videos, designing questions to consolidate their understanding, and a unique information platform. The above improvements facilitated the utilization of SDM for renal biopsy (81.27% in 2020 and 100% in 2021). Even with higher bleeding complications in 2019 and 2020, patients remained satisfied when we launched SDM prior to renal biopsy. Conclusion: This is the first study regarding SDM on renal biopsy. Through SDM, patients had time to understand renal biopsy, including risk and benefit. We recommend SDM to elaborate renal biopsy in clinical practice.

## 1. Introduction

Renal biopsy can offer a detailed diagnosis of renal disease. Pathological findings offer nephrologists accurate diagnosis and disease chronicity. Renal biopsy has been widely used in clinical practice for >100 years [1]. The findings of renal biopsy provide as much as 60% of the information for clinicians’ judgement [2,3,4,5,6]. In another report, kidney biopsy for nephrotic syndrome also affected the management decision in 86% of cases [2]. Despite this, renal biopsy is still not widely used due to many concerns. Factors include the staff’s experience in operating the invasive procedure and its potential complications (in particular, bleeding). Bleeding is the primary concern even with the careful selection of patients [7]. A systemic review of 87 studies (on 118,604 renal biopsies) [8] revealed rates of 3.5% macroscopic hematuria, 11% perinephric hematoma, and 1.6% requiring blood transfusion. Our institute (Taichung Veterans General Hospital, TCVGH) had the largest numbers of renal biopsy in Taiwan according to National Renal Biopsy Registry—a publication of Taiwan Society of Nephrology [9] and the Taiwan Renal Registry Data System (TWRDS) database [10]. Until September 2021, we had performed 8765 renal biopsies, with the lowest bleeding rate nationwide [11,12]. Nevertheless, we are still concerned about bleeding issues in our clinical practice. Therefore, we continuously develop better protocols for communication between patients and doctors to avoid patients’ adverse feelings related to biopsy-related complications and minimize their medical legal actions.

The relationship between patients and doctors is worsening in recent years. Doctor–patient communication is crucial to healthcare [13]. There are three basic models of the doctor–patient relationship [13], including the active-passive model, guidance-cooperation model, and mutual participation model. The traditional model of the physician–patient relationship (authoritative doctor versus passive patient) is no longer applied in today’s clinical practice. Shared decision making (SDM) is a patient–clinician collaboration in making medical decisions that incorporate the patient’s values and preferences alongside the best available evidence [14,15]. It also puts the patient at the center of care [14,15]. In a USA large-scale survey between 2002 and 2014, the mean SDM composite score increased from 4.4 to 5.0 (*p* < 0.01), indicating more patients had perceived SDM [16]. Patient–provider communication that fosters SDM is associated with better chronic disease management [17]. SDM encouraged in clinical practice is improving patient satisfaction [18]. However, SDM has not been used for patients indicated with renal biopsy.

In 2017, our institute (TCVGH) began a multifaceted hospital-wide quality improvement (QI) project for a better physician–patient relationship by using SDM. Better communication on renal biopsy using SDM has not been studied. Here, we aimed to share our experience from the QI projection of SDM on renal biopsy, including the establishment of a core change team, a stakeholder mapping, an improvement framework and an outcome analysis. We also shared the whole process of this QI project and our entire materials of this SDM.

## 2. Materials and Methods

We presented the whole process of our QI collaborative efforts regarding the improvement of communication between patients and doctors on renal biopsy. We also described and accessed projection outcomes. In 2017, we started a QI initiative to reform SDM for renal biopsy in our department. We collected data over 4 consecutive years (2017–2020), including case numbers, complications of renal biopsy (gross hematuria, hematoma, major bleeding, blood transfusion, and transarterial embolization), cases of performing SDM, and satisfaction index based on a questionnaire. The major bleeding included blood transfusion and transarterial embolization, as reported previously [11,19]. All methods were carried out in accordance with relevant guidelines and regulations in TCVGH (Institutional. Review Board number: CE21168A).

### 2.1. Enlisting a Core Change Team

**Figure 1:**
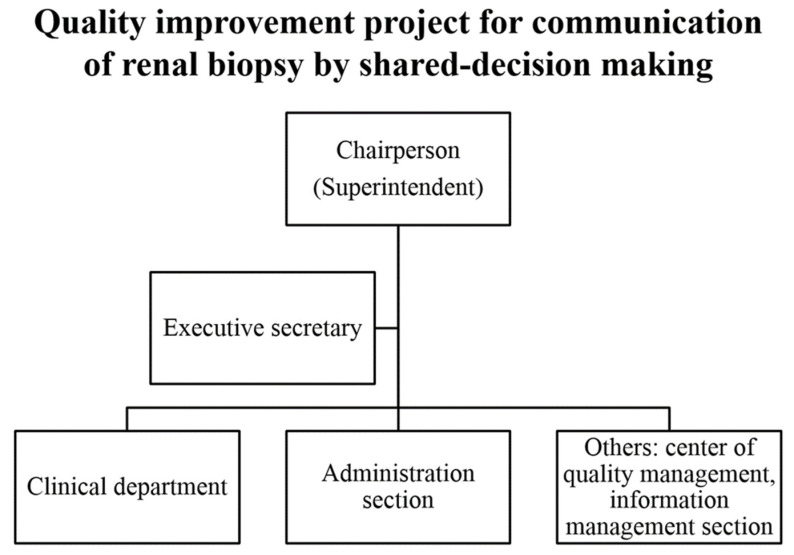
Core team of quality improvement projection for communication of renal biopsy by shared decision making.

First, we enlisted a core change team for this QI project (Figure 1). Its chairperson was the hospital superintendent. In 2017, all TCVGH departments were to start at least one SDM for communication improvement for their own clinical practice. Each SDM was performed by a clinical department with assistance from the administration sector, center of quality management, and information management sector. In the department of nephrology, we chose renal biopsy for the topic of SDM. We proposed that this QI project of SDM can help us achieve better communication between physicians and patients. We also hoped that this project could help us to have a high degree of satisfaction.

### 2.2. Stakeholder Mapping

**Figure 2:**
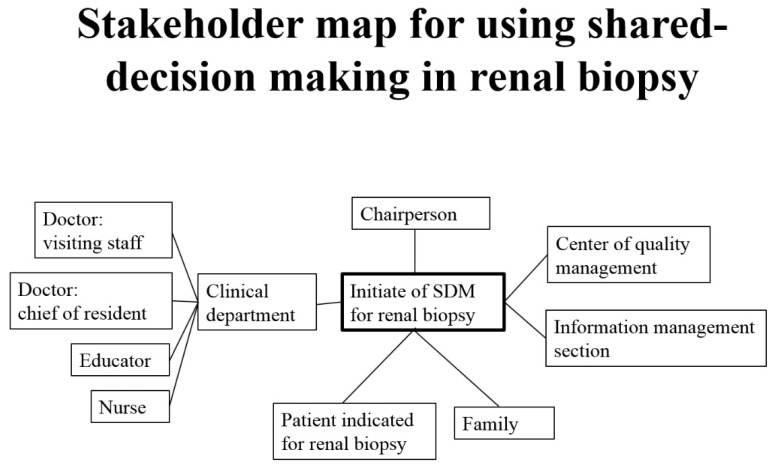
Stakeholder map for using shared decision making in renal biopsy.

After confirming the core team, we used a stakeholder map to identify those who could affect the QI project (Figure 2). We segregated stakeholders into different groups (such as doctor, nurse, educator, patient, and family) and outlined their inter-group relationships. At the center of the map, was the element of quality. The team leader in chief was the hospital superintendent, and leader for renal biopsy QI was the head of nephrology. Specialists of Quality Management and executive secretaries were technical experts with full knowledge of all components of QI. Team members came from different areas of the healthcare system, but doctors and patients/caregivers played the central role.

## 3. Results

### 3.1. Standard Operation Procedure (SOP) of SDM for Renal Biopsy

**Figure 3:**
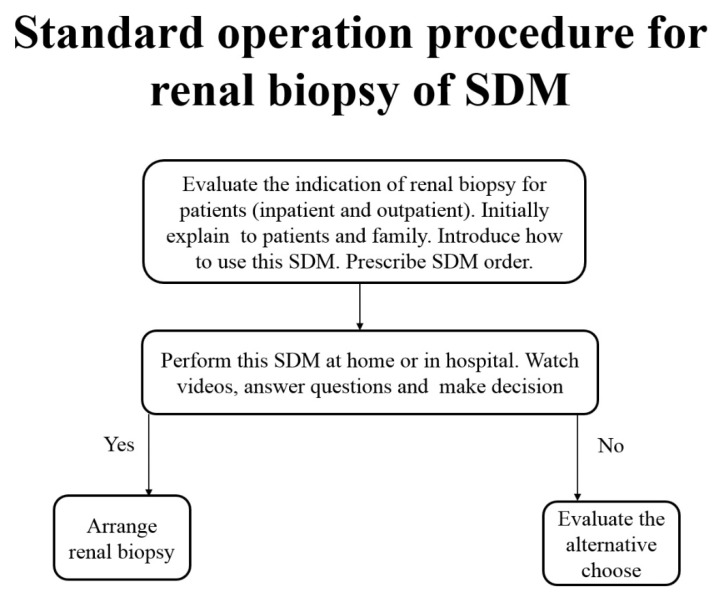
Standard operation procedure for renal biopsy of shared decision making.

We discussed with all associated stakeholders about how to perform SDM for renal biopsy. The SOP in our institute is shown in Figure 3. Most patients indicated for renal biopsy were outpatients. Once fitting the indication of renal biopsy, nephrologists briefly talked to patients and their accompanying family about renal biopsy. The indications are as follows: nephrotic syndrome, nephritis syndrome, or unexplained acute kidney injury. We rarely performed protocol biopsy without indications for allograft kidneys. They then introduced patients and family about SDM, including patient decision aid (PDA). After the visiting outpatient department, patients and their family returned home with ample time to fully understand the introduction they received on renal biopsy, benefit and risk of renal biopsy, and other possible alternative managements. They had to answer five preset questions at home to make sure they fully understood renal biopsy, the risk and benefit of renal biopsy, and other alternative choices. They subsequently made the decision to receive or not receive the renal biopsy. Finally, they completed a satisfaction questionnaire about their feelings about this PDA for renal biopsy. All these procedures were performed by patients and their family at home with ample time. At the next visit to the outpatient department, the nephrologist reviewed their answers in the questionnaire and discussed further with patients and their family. Some of the SDM for renal biopsy started for inpatients, who had emergent problems and were referred for admission. In such an event, nephrologists started the SDM for renal biopsy during the daily ward run. During the few days after admission, patients and their family had ample time to understand renal biopsy and to make their decision. We checked their final answer and discussed it with them whenever necessary.

### 3.2. Improvement of SDM Order System to Facilitate the Utilization

**Figure 4:**
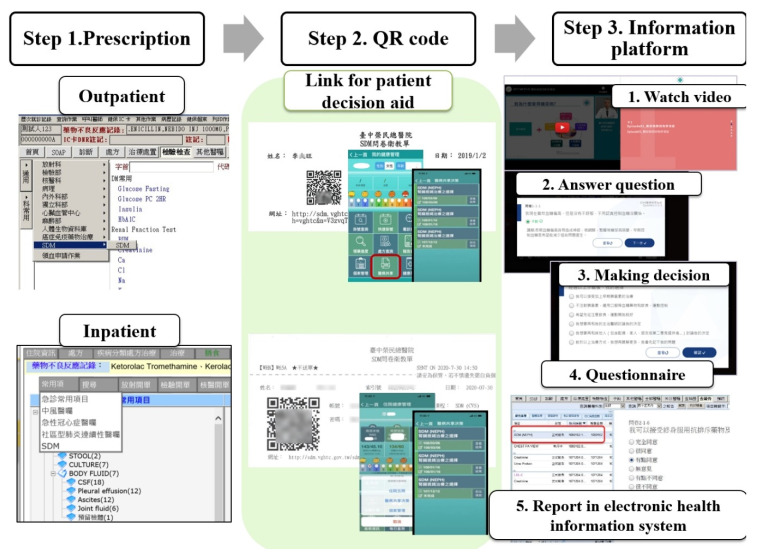
Three steps for shared decision making of renal biopsy.

**Figure 5:**
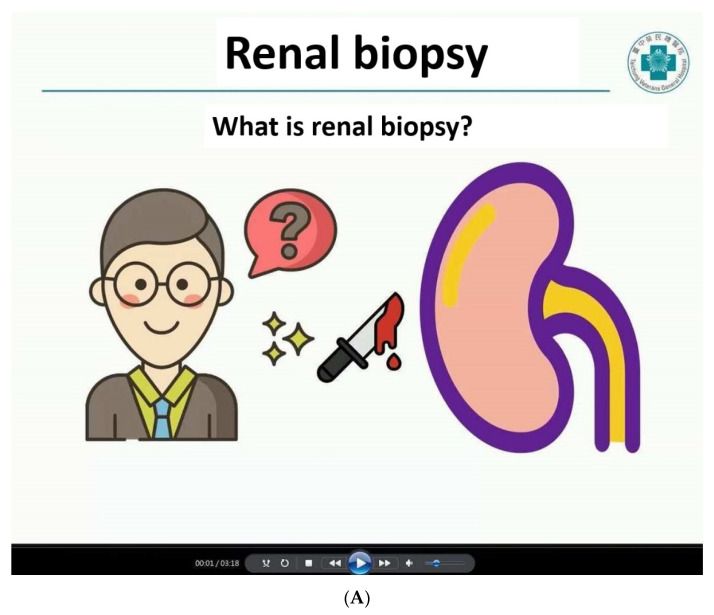

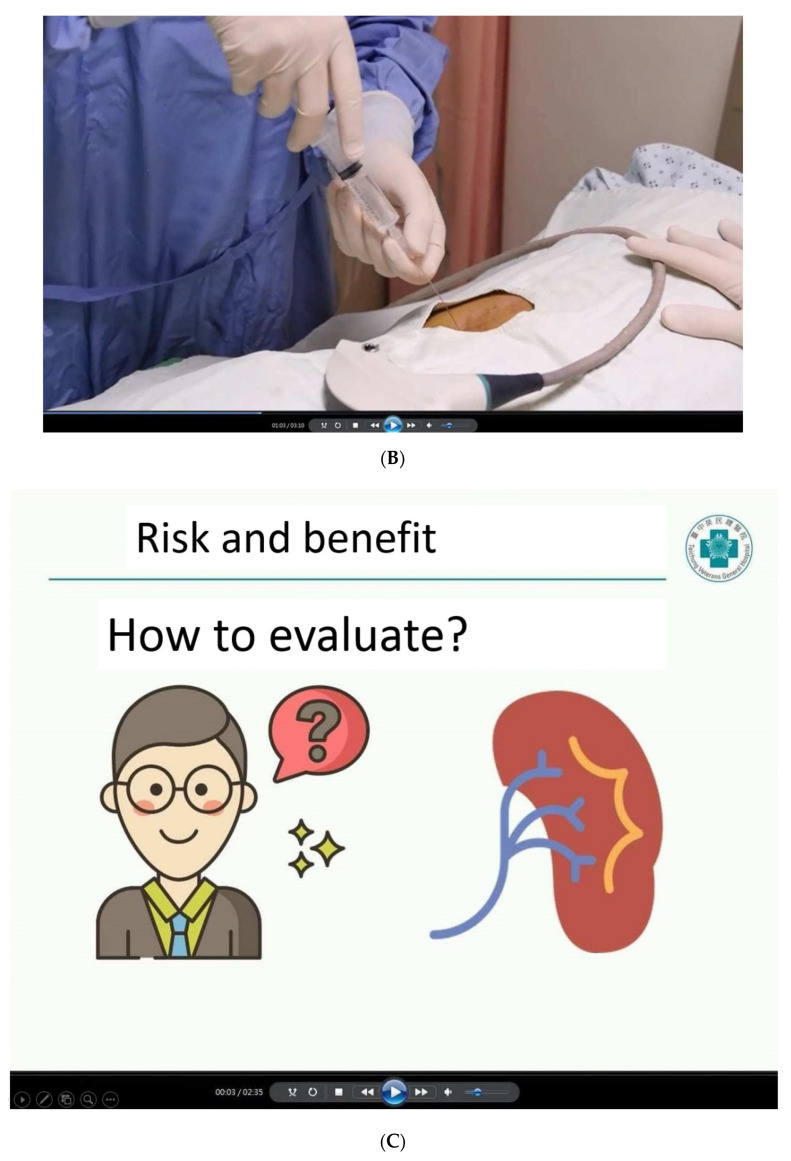

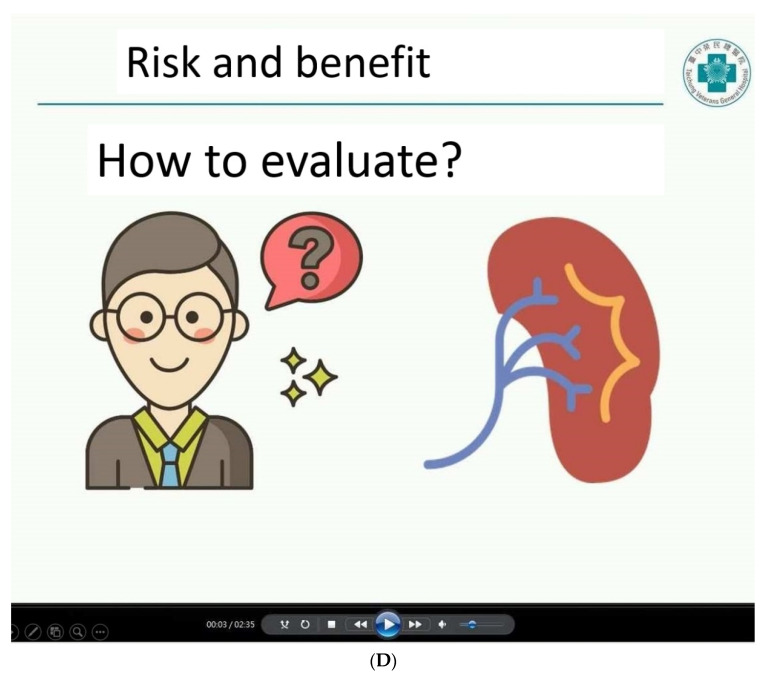
Patient decision aid (video). Duration of video: 3 min and 18 s. (**A**) The introduction of renal biopsy. Duration of video: 3 min and 10 s. (**B**) Live video for renal biopsy. Duration of video: 2 min and 35 s. (**C**) Benefit and risk of renal biopsy. Duration of video: 3 min and 18 s. (**D**) Other alternative choices. Duration of video: 2 min and 36 s.

In 2017, we began our SDM for renal biopsy. In the small and pilot trial for SDM, the PDA was paper based. The satisfactory rate in the questionnaire was high, up to 95.5% (Table 1). In 2018 and 2019, TCVGH further improved all SDMs (Figure 4). First, the center of quality management requested all clinical departments to integrate all SDMs into the institute’s Electronic Hospital Information System (EHIS). Once our clinicians wanted to initiate SDM for renal biopsy, which could be ordered in both outpatient and inpatient settings as regular clinical practice, no extra-loading was added to nephrologists, nurses, and educators. This integrated SDM order system facilitated users. After the first step, patients received their own QR codes, which were linked to videos as PDA (Figure 5). All PDAs were paper free and video-based for better understanding. When patients returned home, they (or helped by a younger family member) simply scanned their unique QR code to watch four videos, including the introduction to renal biopsy (Figure 5A), a live video for renal biopsy (Figure 5B), the benefit and risk of renal biopsy (Figure 5C), and other alternative choices (Figure 5D). In step 3, they were linked to the information platform to watch the above videos. Each of the four videos was ~3 min to avoid watching for a long time. After each video, patients had to answer preset questions to ensure their understanding. Finally, they made the biopsy decision and completed the satisfaction questionnaire. Lastly, their responses to questions were shown in EHIS, and we clinicians were able to review their reports easily.

### 3.3. Better Communication after SDM for Renal Biopsy

The annual case numbers of renal biopsy, SDM, and complications are shown in Table 1. The yearly case numbers of renal biopsy exceeded 200 within the past 5 years. By 30 September 2021, the cumulative case number of renal biopsy reached 8756 in our hospital. The annual rates of gross hematuria were all ~2%, except for 4.6% in 2020. Similarly, rates of hematoma were all <5%, except for 9.6% in 2018, and 11% in 2020. Incidence rates of major bleeding requiring blood transfusion were the highest in 2019 (both 8.5%). SDM for renal biopsy began as a small and pilot trial in 2017 (only 22 cases). After integration into our EHIS order system, the percentage of SDM for renal biopsy went up to 81.27% in 2020 and 100% in 2021. For patients, their satisfaction remained high (100% for the last 3 years) despite high percentages of complications in 2019 and 2020. In 2020 and 2021, their satisfaction rates in the standard approach (non-SDM approach) were 80% and 78%, respectively.

## 4. Discussion

The term “SDM” was first coined in 1972 by Robert Veatch in an ethics report of medicine [20]. It has been widely used to improve patient–clinician relationships recently. The justifications of SDM for renal biopsy are as follows [15,21]. First, unpredictable complications of renal biopsy are a concern to patients. Prior to renal biopsy, a medical history check (especially anticoagulation-related medication and renal size), a physical examination (blood pressure), and selected laboratory tests (coagulation test) are performed to determine the risks of a patient regarding bleeding [22]. Even with meticulous pre-biopsy surveys, post-biopsy bleeding remains unavoidable. The rate of severe bleeding was around 1.96% according to literature reviews [8]. In our institute, SOP for a routine pre-biopsy survey (with checklist and time-out) have been done for years, and the rate of severe bleeding is 0.74% [11], which is the lowest in Taiwan [11,12]. Despite this, we are still concerned about major complications and the potential risk of nephrectomy. Therefore, by using SDM for renal biopsy, patients are allowed ample time to understand the risks and benefits of this invasive procedure. They understand the risk of post-biopsy bleeding, despite the low chances. The bleeding rates, for some unknown reason, increased in 2019 and 2020. SDM for renal biopsy still produced high satisfaction in our patients (100% satisfaction). This can be explained by good communication between doctors and patients via SDM. This can also reduce the rate of legal problems in clinical practice. SDM for renal biopsy in our institute worked very well. Second, more details and clearer briefing on renal biopsy can be done through SDM. To explain renal biopsy, we just show patients with pictures. Using PDA, people can be better informed, more active in their care, and receive care that is more consistent with their values [23]. In 2020, we upgraded our PDA from paper to live videos. Patients can watch the whole procedure of a renal biopsy with no time limitation. Aging patients can also watch videos with their young family to have a better understanding. There is no time or space limitation. Third, enough time for patient consideration is important for SDM renal biopsy. Under the national health insurance system, our doctors are easily accessible for patients. Hence, it is common for a doctor to have 50 patient visits in a morning in Taiwan. Each patient typically receives less than five minutes of attention from the doctor during consultation [24]. This time is too short to allow for a proper explanation of this invasive procedure (renal biopsy). Using SDM, this problem of insufficient time for Taiwanese patients is overcome. We can explain the advantages and disadvantages of renal biopsy much more efficiently via SDM. Initially, nephrologists complained about these unfamiliar ways of communication. However, after beginning SDM for renal biopsy, all physicians in our department agreed that this is a smart and efficient tool for doctor–patient communication. Fourth, without renal biopsy, patients can still receive treatment. Renal biopsy provides more information for diagnosis and treatment. Without such pathological findings with solid evidence, diagnosis may need to depend primarily on clinical experience, taking medical history, and laboratory data. Patients can still choose other medications (such as immunosuppressants) once they fully understand the risk and benefit of these alternative treatment options. In summary, we highly recommend using SDM for the explanation of renal biopsy. With renal biopsy, nearly 50% of patients received different treatments for renal dysfunction.

Below are some related statistics of our hospital. We have the largest numbers of renal biopsy in Taiwan, according to the National Renal Biopsy Registry published by the Taiwan Society of Nephrology [9] and in the Taiwan Renal Registry Data System (TWRDS) database [10]. In addition to the highest quantity of renal biopsy, the quality of renal biopsy is still good in our institute. The department of nephrologists is the first institute in Taiwan to have passed the review exercise of kidney disease-specific care (DSC). We were also invited to speak at the ISQua’ 37th International Conference (The International Society for Quality in Health Care (ISQua)) in 2021 to share experiences of our DCS [25]. In Taiwan, DCS is included in the healthcare quality improvement campaign, and was authorized by the Joint Commission of Taiwan. At the ISQua’ 37th International Conference [25], we presented our experiences of quality improvements, including quality improvement to standard care quality, connections horizontally and longitudinally, risk management, investigation of adverse events, and patient safety. In kidney-DSC, we categorize kidneys into six groups, including acute kidney injury and critical dialysis, chronic kidney disease, renal biopsy, hemodialysis, peritoneal dialysis, and renal transplantation. Specifically, we demonstrated our QI for the first time in this field, on the SDM improvements in the patient–clinician relationship. Moreover, in 2021, we were also awarded a symbol of national quality for our renal biopsy in Taiwan. This symbol represents top safety and quality in our country. All of the above achievements relied on SDM for better communications.

## 5. Conclusions

Our QI program for SDM improved patient–doctor communication for renal biopsy. With the appropriate design and utilization of SDM, even after experiencing post-biopsy bleeding, patients still felt satisfied. We strongly recommend SDM for renal biopsy in our clinical practice.

## Figures and Tables

**Table 1:** Case numbers of renal biopsy and shared decision making and timing according to time period.

	2017	2018	2019	2020	2021 (until 30 September)
Case number of renal biopsy per year	242	251	224	219	172
Accumulative case numbers since	7867	8109	8360	8584	8756
Complication					
Gross hematuria due to biopsy	5 (2.1%)	6 (2.4%)	6 (2.7%)	10 (1.6%)	3 (1.7%)
Hematoma due to biopsy	12 (5.0%)	24 (9.6%)	82 (3.6%)	24 (11.0%)	8 (4.7%)
Major bleeding (n, %)	6 (2.5%)	8 (3%)	19 (8.5%)	2 (0.9%)	2 (1.2%)
Blood transfusion due to bleeding (n, %)	6 (2.5%)	7 (2.8%)	19 (8.5%)	2 (0.9%)	2 (1.2%)
Tran-arterial embolization due to bleeding (n, %)	0	1 (0.4%)	0	0	0
Feeling satisfied (%) from satisfaction questionnaire	95.5%	Unknown	100%	100%	100%
SDM (n, %)	(22, 55%) *	Unknown	5, 2.2%	178, 81.27%	172, 100%
Remark	Pilot trial of SDM by paper: May 2017–August 2017	Temporarily stop SDM by paper. Improve PDA from paper to EHIS and video	Start from eHis system since December 2019		

* 2017.05–2017.05: a total of 40 renal biopsies, of which 22 completed SDM.

## Data Availability

No additional data.
